# Abortion in conflict zones: moral responsibilities of humanitarian agencies in providing safe abortion care

**DOI:** 10.3389/frph.2026.1767552

**Published:** 2026-04-01

**Authors:** Collins Chibueze Anokwuru, Onyeka Chukwudalu Ekwebene, Moses Ifeatu Nwuzoh, Gabriel Chidera Edeh, Chioma Adaora Nwalieji, Ephraim Ikpongifono Udokang, Chidera Gabriel Obi, Stanley Eneh

**Affiliations:** 1 Department of Public Health, Federal University of Technology Owerri, Owerri, Nigeria; 2 Corona Management Systems, Abuja, Nigeria; 3 Youth in Research Hub, Enugu, Nigeria; 4 Department of Pediatrics, Ryan White Center for Pediatric Infectious Disease and Global Health, Indiana University School of Medicine, Indianapolis, IN, United States; 5 Institute of Social Sciences, Sheffield Hallam University, Sheffield, United Kingdom; 6 Department of Haematology/Blood Transfusion, Babcock University Teaching Hospital, Ilishan-Remo, Ogun State, Nigeria; 7 Faculty of Clinical Sciences, University of Nigeria Teaching Hospital Enugu State, Enugu, Nigeria; 8 Faculty of Pharmacy, University of Uyo, Uyo, Akwa Ibom State, Nigeria; 9 Department of Parasitology and Entomology, Nnamdi Azikiwe University Awka, Awka, Nigeria; 10 Department of Community Health, Obafemi Awolowo University, Ile-Ife, Osun, Nigeria; 11 Interdisciplinary Health Sciences, The University of Texas at El Paso, El Paso, TX, United States

**Keywords:** abortion services, conflict zones, health, human right, moral ethics, war

## Abstract

Unsafe abortion remains a leading cause of maternal mortality globally, disproportionately affecting women in conflict zones where health systems are fractured, legal protections are weakened, and access to reproductive care is severely limited. Armed conflict increases the incidence of unwanted pregnancy through sexual violence, displacement, and disruption of contraceptive services, while also heightening barriers to timely, comprehensive abortion care. Despite growing international recognition of abortion as a life-saving intervention, humanitarian agencies often face legal, cultural, and operational barriers that hinder service provision. From an ethical standpoint, obligations grounded in humanity, beneficence, respect for autonomy, impartiality, and the right to health support the inclusion of safe abortion as an essential, reproductive health service. International practice shows that agencies can deliver services through adaptable modalities such as mobile clinics, trained local providers, telemedicine guidance, and integration of the minimum initial service package at onset of crises; but legal variability and criminalization complicate implementation and expose both patients and providers to risk. Operational challenges include disrupted supply chains for medical abortion drugs and equipment, workforce shortages and ethical tensions among staff, monitoring and accountability constraints, and community resistance shaped by cultural and religious norms. The analysis highlights pragmatic strategies that preserve rights while navigating constraints: prioritizing clinical training and values-based preparation for personnel, ensuring availability of evidence-based supplies, embedding timely abortion care within emergency service packages, engaging communities to reduce stigma, and strengthening monitoring systems to protect patients and providers. Safe abortion services in humanitarian settings are not optional adjuncts but moral imperatives. Humanitarian agencies must move beyond rhetorical support to operationalize rights-centred, evidence-based abortion care despite legal and logistical challenges. Upholding reproductive dignity and preventing avoidable harm in conflict zones requires decisive action: sustained commitment to service delivery, workforce capacity, supply security, community engagement, and accountability mechanisms that together translate ethical obligation into lifesaving practice.

## Introduction

Unsafe abortion continues to be a serious public health concern on a global scale, accounting for an estimated 13% of all maternal deaths, or around 39,000 deaths per year, the majority of which take place in areas with limited resources or that are impacted by war (1). Unsafe abortion is defined by the WHO as a pregnancy termination procedure carried out by someone who is not qualified to do so or in a setting that does not meet basic medical requirements (2). In conflict zones settings, where infrastructure is damaged, health systems are frequently fractured, sexual violence and unintended pregnancy intersect, and legal protections are either halted or unclear, the situation is grimmer.

WHO estimates that around 68,000 women die annually due to unsafe abortion, largely from haemorrhage, infection, or organ damage (3). In countries affected by conflict, maternal mortality ratios can be up to five times higher than in stable regions (4). Reports from Sudhakaran et al. estimate that maternal mortality attributed to abortion may reach 25%–50% in refugee and displacement situations where abortion care is absent or limited (5, 6). Similarly, UNFPA analyses suggest that between 25% and 50% of maternal deaths in some refugee settings are linked to unsafe abortion complications (7). However, estimates of abortion related mortality in conflict and displacement settings vary by data source and methodology and should be interpreted with caution due to underreporting, attribution challenges, and measurement constraints in insecure environments. Despite this uncertainty, abortion related services have historically been neglected in humanitarian responses, reflecting compounded health system disruption during conflict, reduced access to emergency obstetric care, and persistent misconceptions among providers and communities about the restrictiveness of national law (8, 9).

These factors not only constrain access to essential reproductive health care but also contribute to higher levels of unintended pregnancy by increasing exposure to sexual violence, disrupting contraceptive supply chains, and weakening social and legal safeguards (10). In response to these pressures, humanitarian organizations have progressively expanded their sexual and reproductive health mandates to include emergency abortion treatment and post abortion care alongside maternal health and contraception services. The Inter Agency Working Group (IAWG) positions abortion care as a vital and life saving intervention in emergency contexts in the Inter Agency Field Manual on Reproductive Health in Humanitarian Settings (11). However, in practice, safe abortion services remain inconsistently implemented and are sometimes restricted or excluded by humanitarian actors, often due to donor constraints, legal uncertainty, or perceived ethical dilemmas (12). Thus, this paper explores the health infrastructure, legal, and social consequences of restricted abortion access in emergency settings and analyses these challenges in relation to the core humanitarian principles of humanity, neutrality, impartiality, and independence.

## Conflict zones and health infrastructure

Armed conflict systematically destroys health infrastructure and disrupts supply chains. For example, the wars in Syria and the Democratic Republic of the Congo respectively, has left medical supply chains disrupted, clinics and pharmacies looted, and health workers fleeing, so that essential services are collapsing (13, 14). South Sudan's decades of war likewise left its weak health system in ruins, such that only about 25%–30% of the population had any access to care by 2015 (15). In summary, conflict turns hospitals and clinics into ruins, forcing survivors to go without care or seek assistance in makeshift tents and mobile units (13–15).

Conflict also triggers massive social and economic upheaval that harms women's health. Millions are uprooted, as observed in Syria, where more than seven (14) million internally displaced persons live in overcrowded camps or informal sites (13). Displacement brings food shortages, unsanitary conditions, and disease outbreaks, as noted by WHO (13). Physical barriers, including destroyed roads, checkpoints, and the risk of violence, deter travel to clinics, especially in rural or conflict-heavy areas. Economic collapse makes care unaffordable as impoverished families cannot pay for transport or medicines. Social factors such as stigma and restrictive norms may also worsen, which can discourage women from seeking reproductive care, especially abortion or family planning in conservative communities under strain. These combined disruptions leave pregnant women at extreme risk, lacking both food and healthcare, and drive many towards unsafe solutions.

Humanitarian agencies have increasingly stepped in to fill these gaps, expanding their mandate to include sexual and reproductive health (SRH). International guidance now explicitly treats abortion as an essential component of emergency care. For instance, the 2018 Inter-Agency Field Manual (IAFM) for SRH in crisis calls for integrating safe abortion care (SAC) as part of the Minimum Initial Service Package (16, 17). MSF (Doctors Without Borders), since a 2004 policy resolution, has worked to incorporate safe abortion into its services (17). By 2023 MSF teams delivered over 54,600 safe abortions across 37 countries, even as they continue to treat the tens of thousands of complications from unsafe procedures (18). WHO's latest Abortion Care Guideline similarly reaffirms abortion as an essential, evidence-based health intervention with a strong human-rights foundation (19, 20). The United Nations Population Fund (UNFPA) also urges treating abortion as an essential service in emergencies (21). In this way, humanitarian policy has shifted, such that safe abortion care is now seen not as an optional extra, but as integral to preserving women's health and rights in crisis.

In practice, agencies use flexible delivery models to reach women. Mobile clinics and outreach teams are widely used, as reported by UNFPA where 28 mobile gynaecological teams were dispatched across Ukraine, providing SRH services to 111,000 women (22). Telemedicine is another innovation, as demonstrated by MSF, which piloted a hotline in the Middle East, remotely guiding 22 women through medical abortions at home (23). Other approaches include training midwives in manual vacuum aspiration and using the WHO Information, Education, and Communication (IEC) kits for post-abortion care. These programmatic adaptations help circumvent clinic shortages and legal barriers, making SRH care available even in insecure areas.

Agencies generally work through health clusters or agreements with ministries of health to ensure legality and scale. For example, in Bangladesh's Rohingya camps, UNFPA and Ipas collaborated with the government's health system to rapidly set up menstrual regulation (safe abortion) services, which within one month of the 2017 influx, eight health facilities were offering abortions, and by mid-2019 over 300 providers across 37 clinics had been trained (24). In Ukraine, UNFPA partnered with local NGOs (e.g., the Ukrainian Women's Foundation and family-planning charities) to establish 123 “barrier-free” SRH clinics for displaced women (22). In summary, successful programs align with ministries for oversight, train and support local health workers and NGOs, and involve community leaders in outreach, ensuring that even in conflict settings, comprehensive reproductive services, including safe abortion, reach women in need.

## Legal and social landscape of abortion in conflict zones

Conflict zones are known for fragility and displacement, which can put women and children at high risk of unwanted pregnancy, sexually transmitted infections, and other risks of sexual violence and exploitation (25). This fragility and displacement may lead to the violation of international guidelines governing humanitarian health practice, hence raising profound ethical questions for humanitarian actors. This tension necessitates a closer examination of the ethical principles that govern reproductive health interventions in conflict settings, as discussed below.

### Ethical framework and principles

Health interventions in conflict settings are commonly framed as being guided by beneficence and duty of care, requiring action that promotes wellbeing and prevents avoidable harm among affected populations. However, in practice, these principles are not value neutral and often come into tension with legal restrictions, security risks, and institutional caution (26, 27). Respect for autonomy further requires recognition of girls' and women's reproductive decision making, yet autonomy is frequently constrained in humanitarian contexts by coercion, sexual violence, displacement, and service inaccessibility (28). In such settings, appeals to neutrality or legal compliance can function less as ethical safeguards and more as practical justifications for limiting reproductive choice.

Given the well documented association between armed conflict, sexual violence, and unintended pregnancy, restricting access to comprehensive reproductive health services, including safe abortion, raises serious ethical concerns about preventable harm and discriminatory exclusion (28, 29). Normative guidance increasingly reflects this position. The WHO Abortion Care Guideline grounds abortion access within beneficence, non-maleficence, autonomy, and equity, and calls on health systems and humanitarian actors to remove legal, policy, and operational barriers that impede timely care, including in emergencies (19). Similarly, International Federation of Gynecology and Obstetrics (FIGO) situates abortion care within medical ethics, reproductive justice, and gender equality, and argues that conscientious objection must not translate into effective denial of services, placing responsibility on institutions to ensure access through referral or task sharing arrangements (25).

Taken together, these frameworks shift the ethical debate from whether obligations exist to how they should be operationalized under constraint. The central ethical challenge for humanitarian actors is therefore not only to acknowledge duties in principle, but to justify and design implementation choices when legal, political, and security pressures make rights consistent service delivery difficult.

### International human right reproductive right frameworks

Several international human rights guidelines and instruments recognise reproductive health, including safe abortion, as a major component of the right to health, which may be violated in conflict zones. The Geneva Conventions require the treatment of wounded and sick civilians, which is not limited to reproductive health services for survivors of sexual violence (30). Other guidelines, such as the International Covenant on Economic, Social and Cultural Rights (ICESCR), the Convention on the Elimination of All Forms of Discrimination Against Women (CEDAW), and FIGO not only support access to healthcare but also uphold the right to reproductive health autonomy, including access to safe abortion in conflict zones (25, 31, 32).

### Variation in legal restrictions

The legal landscape governing abortion in conflict zones varies considerably across national context. In some countries, abortion remains criminalised except to save the pregnant woman's life, while others have no exceptions (33). This not only increases the risk of unsafe abortion but also poses a challenge to healthcare workers in those settings as to whether to respect local laws or uphold international human rights norms (34, 35). However, humanitarian organisations working in conflict zones should maintain a balance by respecting local laws while offering safe abortion care in accordance with what the law permits (35).

### Social, cultural and religious dimensions

In many societies, the acceptability and accessibility of abortion services are influenced by social stigma, religious doctrine, cultural beliefs, and practices, which may deter women and girls living in such societies from seeking or receiving services, even when these are legally permitted by local law in that conflict zone (36–38). For instance, Palestinian women may prefer to follow Palestinian law due to religious beliefs over the more abortion-permissive Israeli law (39). These social, cultural, and religious influences may trigger backlash against humanitarian service providers in conflict zones, thereby hindering service provision (40).

## Moral responsibilities of humanitarian agencies in providing safe abortion services

### Synthesis of moral imperatives vs. operational realities

The provision of safe abortion services is a complex issue that involves balancing moral imperatives with operational realities. The International Federation of Gynecology and Obstetrics asserts that the right to safe abortion is an integral part of sexual and reproductive health and rights, gender equality, reproductive justice, and universal health care, particularly in cases where pregnancy poses a risk to the life or health of women (41). Nonetheless, abortion care, operation, and prompt provision of non-judgmental services are peremptory, indeed in emergencies. Despite the evidence suggesting the need for safe abortion services and consensus in the humanitarian community about the importance of providing comprehensive abortion care, operational realities such as legal restrictions, social stigma, fear of death from unsafe abortion procedures, and lack of access to healthcare services can limit the availability of safe abortion services (42).

### Balancing neutrality with advocacy for reproductive rights

Healthcare providers must balance their professional obligation to remain neutral with their moral obligation to advocate for reproductive rights. This can be particularly challenging in settings where abortion is heavily stigmatized or restricted (43, 44). However, advocacy for reproductive rights is essential to ensuring that women have access to safe and comprehensive reproductive healthcare services (45). Advocacy is core to the provision of sexual and reproductive health, and it can take many forms, including supporting policy changes that promote reproductive rights, providing accurate information about abortion services, and engaging in public discourse about the importance of reproductive autonomy. By advocating for reproductive rights, healthcare providers can help to promote a culture of respect for women's dignity, their right to personal reproductive autonomy, and collective gender equality (46).

### Implications for humanitarian ethics and global health policy

In humanitarian settings, there is often limited access to safe abortion services, and women may be forced to undergo unsafe abortions and seek care from unqualified providers, resulting in significant mortality and morbidity, mostly in settings where healthcare systems are already overstretched. Therefore, as a critical component of comprehensive reproductive healthcare, Global health policy should prioritize the provision of safe abortion services. This requires an obligation to ensure that abortion services are affordable, accessible, and safe, especially in low-income countries (47). This can help to promote the health and well-being of women and girls, particularly in humanitarian settings.

## Challenges and dilemmas

### Legal and security constraints

Eliminating unsafe abortion is a global priority from an international, public health, reproductive rights, and human rights perspective. Achieving this requires removing social and legal barriers to voluntary termination of pregnancy, while also ensuring that accessible reproductive health services, including safe abortion care, are available. Both governmental and non-governmental humanitarian organizations frequently encounter legal and security constraints that impede their capacity to respond effectively to crises. Abortion is a highly safe procedure when conducted by qualified personnel adhering to current medical protocols; however, it remains legally restricted in numerous countries (48), which can make it worse in war zones. Such restrictions necessitate that abortion be performed illegally, in a clandestine manner, and under unsafe conditions. The imposition of restrictive laws and regulations by governments can inhibit their ability to operate in particular areas or to provide specific services. Furthermore, security threats, including kidnapping, violence, or arrest, can restrict humanitarian workers' access to the affected populations (49, 50).

### Resource limitations and supply chains

A crucial component of abortion care relies on a functional supply chain to ensure the availability of supplies and abortion drugs such as misoprostol or mifepristone, manual vacuum aspiration (MVA) kits, or other key medicines such as analgesics and antibiotics within the health system. Disruptions in the supply of medical abortion drugs delay the provision of abortion services and can increase the risks to the health of women. Furthermore, inefficiencies such as poor funding for procurement and distribution, weak management systems, lack of trained staff, and governmental policies that limit procurement or distribution of supplies create substantial bottlenecks in the supply chain (51).

### Ethical tensions among staff

Healthcare organizations face ethical tensions among workers, particularly in complex and high-pressure environments. A variety of factors undercut the ability of healthcare workers to fulfill their responsibilities, such as cultural differences that can lead to differing values and perspectives on service response, while moral dilemmas such as prioritizing aid to certain groups over others can create ethical tensions. Other issues affecting healthcare worker rights and the roles they ultimately play in health systems include facilities and communities' challenges to their rights, roles, and responsibilities, such as conflicting policies laws, and guidelines; pressure to achieve coverage and quality; violations of the rights and professionalism of healthcare workers, undercutting their ability and motivation to fulfill their responsibilities; inadequate stewardship of the private sector; competing paradigms for decision-making such as religious beliefs, that are inconsistent with professional responsibilities; donor conditionalities and fragmentation; and, the persistence of embedded practical norms that are at odds with healthcare worker rights and responsibilities. The tensions lead to a host of undesirable outcomes, ranging from professional frustration to the provision of a narrower range of services or poor-quality services (52).

### Community resistance and stigma

There is substantial evidence that at the community level, Community Health Workers can deliver a wide range of services, that they can increase access to essential healthcare services, and that scaling up community-level interventions can lead to large improvements in reproductive, maternal, and child health (53). However, the challenges to implement community health services in a humanitarian setting can be daunting, and these include acute and protracted crises, such as prolonged periods of conflict and insecurity and during population displacement, reduced access to services when travel was limited, security threats and psychological trauma because of their work (54).

### Monitoring, evaluation, and accountability

Collecting and analyzing data is crucial to understanding the impact of programs and making informed decisions. In addition, establishing accountability mechanisms ensures transparency and accountability to affected populations. Challenges in monitoring, evaluating, and ensuring accountability in programs are being faced by healthcare organizations. Such challenges include criminalization of abortion practices, poor quality of care, and reduced access to safe abortion. However, these challenges must be navigated to provide effective and efficient aid to those in need. Recognizing these challenges does not negate ethical responsibility; rather, it underscores the need for deliberate, evidence-informed strategies that can reconcile moral obligations with operational realities that can better address the needs of affected populations (55).

## Strategic recommendations

In order to protect their rights, humanitarian agencies and systems must ensure that women, girls, and professionals are not criminalized for either seeking or providing timely and necessary abortion treatment and services (25). This can be achieved through (Table 1).

1. **Advocacy for Policy Reform on Abortion Access**: Humanitarian agencies must intensify efforts to advocate for the decriminalization and legalization of abortion, particularly in conflict-affected countries. Strategic policy engagement with United Nation (UN) bodies, government authorities, ministries of women affairs and human right organizations is necessary to **eliminate the legal, security and social constraints to voluntary termination of pregnancy,** and enable safe, timely, and equitable abortion services in humanitarian settings.
2. **Skilling up the Healthcare Workforce**: Health care providers who work in emergency situations ought to receive training on how to deliver safe, high-quality abortion services that respect patients' rights. Almost always required, workshops that cover attitudes, values, and beliefs are best conducted prior to technical training and service delivery. Long before a crisis, UN agencies, governments, and international and national NGOS, among others, must support the capacity of local Ministries of Health and Disaster Management, national and community-based organizations, health workers, and communities themselves to strengthen health systems with an emphasis on comprehensive reproductive health, accessibility, and resilience-building within a rights-based framework. Operational readiness, training, and supportive supervision enable abortion service delivery even in complex emergency environments.
3. **Timely Access Medical Equipment and Medications:** Time-sensitive critical health care is a fundamental human right. This right to health and scientific advancement should not be denied because of a person's circumstances; even in an emergency, refugees, asylum seekers, and displaced individuals should have access to safe abortion (56). In crisis setting, affected populations should be informed about the abortion services that are accessible and the circumstances in which they can be rendered, as well as the availability of context-relevant and evidence-based equipment and drugs. Moreover, Casey et al. (59) and Krause et al.'s (60) studies highlight the urgent need to strengthen commodity management processes to prevent stock-outs and provide consistent access to care.
4. **Integration of Minimum Initial Service Package (MISP)**: The MISP is an emergency response that should be put into place as soon as a humanitarian crisis occurs. Its purpose is to provide women and girls with the minimal sexual and reproductive health (SRH) interventions required to prevent mortality and morbidity (57). Pre-crises planning and routing MISP audits can mitigate the occurrence of delayed or partial implementation.
5. **Community Engagement and Stigma Reduction**: To establish a supportive environment for women seeking abortion services in conflict areas, government and non-governmental organizations should enter a memorandum of understanding (MOU) with community leaders, survivors, medical professionals, law enforcement, and other stakeholders (58). Additionally, community members ought to be informed and trained against stigmatizing those who obtain abortion services. Leaders in the community should incorporate anti-stigmatization customs to help women who deserve abortion services feel less afraid.

**Table 1:** Linking evidence to strategic recommendations for safe abortion care in humanitarian settings.

Recommendation	Supporting evidence/examples	Implementation preconditions (Who, what, How)	Key risks	Mitigation strategies	Suggested indicators
Advocacy for Policy Reform on Abortion Access	Restrictive abortion laws in conflict settings force clandestine, unsafe procedures, increasing maternal morbidity and mortality. (46, 48–50)	Who: Humanitarian agencies, UN bodies, ministries of health, human rights organizations What: Policy dialogue, legal reviews, protection clauses for providers and patients. How: Strategic advocacy, engagement with ministries of women affairs, legal aid partnership.	Political backlash or suspension of humanitarian access	Frame advocacy within human rights, lifesaving care, public health and humanitarian law. Use incremental reforms and coalition advocacy	Humanitarian permits for abortion care; Policy inclusion; legal clarifications; Reduction in reported providers arrest or service disruption
Skilled Training for Healthcare Providers	Prepared and supported providers can deliver care even in restrictive environments (52).	Who: Ministries of Health, NGOs, professional associations. What: Values clarification, clinical abortion training, supportive supervision. How: Pre-crisis capacity building; in-service training; mentorship models embedded in emergency preparedness plans; referral pathways	Conscientious objection; staff resistance; burnout due to moral conflict	Values clarification; Ongoing ethical support; peer mentoring; non-punitive work environments; task-sharing	Provider coverage, Provider confidence and attitude scores, and referral completion.
Ensure Timely Access to Medical Equipment and Medications	Commodity management failures (51, 56)	Pre-positioning supplies, emergency procurement waivers, integrated logistics management systems of essential abortion drugs and equipment	Stock-outs; border restrictions; Confiscation of supplies.	Clear labeling as essential medicines, diversified supply routes, donor alignment with humanitarian standards	Stock-out frequency; Average time to replenish supplies; Proportion of facilities with full abortion commodity kits
Integration of the Minimum Initial Service Package (MISP)	The MISP is an evidence-based framework proven to reduce preventable maternal morbidity and mortality in emergencies when implemented early (57)	Who: Humanitarian coordinators, health cluster leads, Ministries of Health. What: Full MISP implementation including abortion-related components. How: Immediate activation at crisis onset, standardized protocols, coordination through health clusters.	Delayed activation or partial implementation.	Pre-crisis planning, accountability within health clusters, routine MISP audits.	Time from crisis onset to MISP activation; Inclusion of abortion care in MISP plans; Facility-level MISP compliance
Community Engagement and Stigma Reduction	Engagement with community leaders and CHWs improves acceptability and service uptake (53, 54, 58).	Dialogue forums, CHW training, culturally sensitive messaging.	Community backlash or targeting of providers/clients.	Anonymized reporting; Confidential service models, trusted intermediaries, gradual norm-shifting approaches	Complication rates; Service uptake trends;

### Limitations of the study

First, as a Policy and Practice perspective review, it does not provide the exhaustive coverage of a evidence synthesis or empirical findings. Most of the evidence is context-specific, shaped by national legal frameworks, security conditions, displacement patterns, and baseline health system capacity, limiting generalizability across conflict typologies. Second, epidemiological data on abortion-related outcomes in conflict settings remain limited due to underreporting, stigma, and challenges in attributing maternal deaths. Finally, key evidence gaps remain, including comparative effectiveness of service delivery models, legal risk mitigation strategies, standardized monitoring indicators, and long-term outcomes of stigma-reduction interventions. Addressing these gaps should be a priority for future humanitarian research.

## Conclusion

It is evidently clear that unsafe abortion remains a leading cause of preventable maternal mortality in crisis settings, exacerbated by fractured health systems, legal ambiguity, and sociocultural stigma. Yet, safe abortion is a medically simple, cost-effective, and essential component of emergency health response. This review has demonstrated that humanitarian agencies bear a moral and professional responsibility to ensure a safe abortion services, grounded in international human rights law, medical ethics, and the core humanitarian principles of humanity, impartiality, neutrality, and independence. Ultimately, the provision of safe abortion in conflict zones is not merely a clinical obligation; it is a moral imperative. Evidence from humanitarian responses indicate that when appropriately integrated into emergency reproductive health programming, safe abortion services can be delivered in ways that are context-sensitive, right-informed and operationally feasible. Humanitarian actors may benefit from moving beyond rhetorical support and operational hesitancy to embrace abortion care as a key component of comprehensive emergency health response for women. Aligning ethical commitments with practical implementation has the potential to support reproductive justice, protect vulnerable populations, and fulfill their ethical mandate to do no harm.
